# Visual Field Reconstruction in Hemianopia Using fMRI Based Mapping Techniques

**DOI:** 10.3389/fnhum.2021.713114

**Published:** 2021-08-10

**Authors:** Hinke N. Halbertsma, Holly Bridge, Joana Carvalho, Frans W. Cornelissen, Sara Ajina

**Affiliations:** ^1^Laboratory for Experimental Ophthalmology, University of Groningen, University Medical Center Groningen, Groningen, Netherlands; ^2^Wellcome Centre for Integrative Neuroimaging, Nuffield Department of Clinical Neurosciences, University of Oxford, Oxford, United Kingdom; ^3^Champalimaud Centre for the Unknown, Champalimaud Foundation, Lisbon, Portugal; ^4^Department of Neurorehabilitation, National Hospital for Neurology and Neurosurgery, London, United Kingdom

**Keywords:** cortical blindness, visual cortex, residual vision, population receptive field, visual field (VF)

## Abstract

**Purpose:**

A stroke that includes the primary visual cortex unilaterally leads to a loss of visual field (VF) representation in the hemifield contralateral to the damage. While behavioral procedures for measuring the VF, such as perimetry, may indicate that a patient cannot see in a particular area, detailed psychophysical testing often detects the ability to perform detection or discrimination of visual stimuli (“blindsight”). The aim of this study was to determine whether functional magnetic resonance imaging (fMRI) could be used to determine whether perimetrically blind regions of the VF were still represented in VF maps reconstructed on the basis of visually evoked neural activity.

**Methods:**

Thirteen patients with hemianopia and nine control participants were scanned using 3T MRI while presented with visual stimulation. Two runs of a dynamic “wedge and ring” mapping stimulus, totaling approximately 10 min, were performed while participants fixated centrally. Two different analysis approaches were taken: the conventional population receptive field (pRF) analysis and micro-probing (MP). The latter is a variant of the former that makes fewer assumptions when modeling the visually evoked neural activity. Both methods were used to reconstruct the VF by projecting modeled activity back onto the VF. Following a normalization step, these “coverage maps” can be compared to the VF sensitivity plots obtained using perimetry.

**Results:**

While both fMRI-based approaches revealed regions of neural activity within the perimetrically “blind” sections of the VF, the MP approach uncovered more voxels in the lesioned hemisphere in which a modest degree of visual sensitivity was retained. Furthermore, MP-based analysis indicated that both early (V1/V2) and extrastriate visual areas contributed equally to the retained sensitivity in both patients and controls.

**Conclusion:**

In hemianopic patients, fMRI-based approaches for reconstructing the VF can pick up activity in perimetrically blind regions of the VF. Such regions of the VF may be particularly amenable for rehabilitation to regain visual function. Compared to conventional pRF modeling, MP reveals more voxels with retained visual sensitivity, suggesting it is a more sensitive approach for VF reconstruction.

## Introduction

The visual field (VF) is the region of the world that we can perceive and in the healthy human binocular visual system the VF subtends more than 200° (Spector, 1990), allowing us to approximately monitor half of the scene around us at any one instance. Damage to either the eyes or to the brain can reduce the field of view that can be perceived. When the VF is reduced due to retinal damage to one eye, the other can cover much of the region of lost function. However, when damage occurs in the visual pathway beyond the optic chiasm, the representation of one half of the VF is lost in both eyes, known as homonymous hemianopia (or hemianopia for short).

Standard methods for objective VF examination, i.e., standard automated perimetry (SAP), rely on the participant consciously reporting the presentation of visual stimuli (most commonly small points of light of differing intensity) at static and fixed locations. This type of examination in an observer with hemianopia will typically reveal a partial or full loss of unilateral VF contralateral to the lesioned hemisphere. However, this type of test, while critical for practical issues such as suitability for driving, does not reveal any non-conscious vision that is often present in patients with hemianopia (Stoerig and Cowey, 1989, 1992, 1997; Weiskrantz, 1993, 1996; Tomaiuolo et al., 1997; Tamietto and de Gelder, 2008; Ajina et al., 2015; Ajina and Bridge, 2018, 2019; Danckert et al., 2019; Sanchez-Lopez et al., 2020). The definition of regions of the VF as “blind” based on perimetric measures additionally does not reflect the finding that stimulation within this blind field can lead to neural activity in the visual areas of the brain (Barbur et al., 1993; Morland et al., 2001; Bridge et al., 2008; Radoeva et al., 2008; Tinelli et al., 2013; Papanikolaou et al., 2014; Schneider et al., 2019; Sanchez-Lopez et al., 2020). Disadvantages of SAP therefore consist of its low spatial specificity, the high performance and attention required by the participant, the fact that it only captures conscious vision and its limited flexibility in testing stimuli.

Functional magnetic resonance imaging (fMRI) techniques that involve systematic stimulation of the VF with highly salient visual stimuli can provide an alternative method to determine regions of the VF that lead to neural activation. In particular, population receptive field (pRF) mapping provides the ability to determine both the location and size of neural responses within the VF (Dumoulin and Wandell, 2008), an approach that has now been used extensively in the healthy and diseased human visual system (Wandell and Smirnakis, 2009; Haak et al., 2012; Dumoulin and Knapen, 2018; Silson et al., 2018; Ahmadi et al., 2019; de Best et al., 2019; Halbertsma et al., 2019). More specifically, Papanikolaou et al. (2014) and Haak et al. (2014) measured pRF size and location in primary visual cortex in a small number of patients with hemianopia, and concluded that there was very little plasticity following damage. A later paper revealed that it was also possible to map pRFs in human motion area hMT+ within the blind field, indicating that these responses are not sufficient to induce conscious vision (Papanikolaou et al., 2019). Interestingly, a very recent study investigating the effect of perceptual training in hemianopia showed that regions of the VF that exhibited neural activity prior to training were more likely to show improved visual function on perimetry after training (Elshout et al., 2021). Therefore, fMRI based mapping may overcome the disadvantages of SAP, as it allows for high-spatial frequency stimulation of the VF, requires passive viewing by the participant and is flexible regarding the testing stimuli that can be used (for example: Alvarez et al., 2015; Zuiderbaan et al., 2017; Yildirim et al., 2018), and may allow for capture of visual processing that remains uncaptured by SAP.

Understanding where there is neural tissue that still responds to visual stimulation is important for targeting visual rehabilitation programs, since many use relatively small stimuli. Therefore, it would be useful to employ an approach that can indicate a map of the VF that reflects responsive neural tissue without requiring participants to make decisions about whether a particular stimulus was present or absent and with high spatial specificity.

Here we probed the potential of two different pRF-based mapping techniques to allow reconstruction of the VF in people with hemianopia. One is a conventional, yet fast, approach (pRF-mapping; Dumoulin and Wandell, 2008) and the other, a more computationally demanding one, known as micro-probing (MP) (Carvalho et al., 2020). While both techniques can reconstruct a VF with good correspondence to a simulated VF defect (Papanikolaou et al., 2015; Hummer et al., 2018; Prabhakaran et al., 2020; Carvalho et al., 2021), MP has the potential to reveal any existing multiple or bilateral VF representations that may underlie unconscious or blindsight capacities in hemianopia (Muckli et al., 2009; Fracasso et al., 2016; Carvalho et al., 2020). Therefore, we hypothesized that both mapping techniques can reconstruct the VF in patients with hemianopia, as determined by perimetry, but may reveal additional responsive regions undetected by perimetry. Furthermore, we expected MP to provide us with a finer-grained representation of the patients’ VF.

## Materials and Methods

### Participants

Thirteen patients (four female) and eight controls (three female) were included in this study. Twelve of the patients had sustained a stroke and one had undergone benign tumor resection involving unilateral damage to the primary visual cortex which resulted in homonymous quadrantanopia or hemianopia. VF examinations were performed on the patients with either the Esterman (one patient) perimetry or the humphreys field analyzer (HFA) using the 24-2 or 30-2 grid and the SITA-Fast or the SITA-Standard. Whereas the Esterman makes a distinction between sighted and blind locations of the VF, the HFA denotes the contrast sensitivity (in dB) at various VF locations where higher values represent higher sensitivity. Blindsight examinations were performed on the patients as part of a previous study (Ajina et al., 2015). Ten of the patients were classified as “blindsight positive” on a motion detection task with a 5° or 8° diameter stimulus. Blindsight was defined as achieving either an average score, or a score for stimuli of 100% contrast, that was significantly above chance using a statistical threshold of *p* < 0.01 and a cumulative binomial distribution. This was despite a perimetry threshold *p* < 0.005 or <−20 dB (whichever was more stringent) for pattern deviation at the stimulus location, compared to age-matched controls. Of the thirteen patients, ten had VF loss that allowed for visual stimulus test locations within 6.5° of central fixation (the region visible in the fMRI scanner). Eight of those patients were blindsight positive, and two were blindsight negative. Written consent was obtained from all participants, who had normal or corrected-to-normal vision, no visual neglect, and no eye or neurological diseases (other than the cause of the hemianopia, in case of patients). Ethical approval was provided by the Oxfordshire Research Ethics Committee B (Ref B 08/H0605/156). Participant demographics are presented in Table 1.

**TABLE 1:** Patient demographics and blindsight status.

Patient	Sex	Age	Visual Field test	Motion Blindsight status	Cause	Time since lesion onset (months)
1	M	29	Esterman	Positive	R. occipital infarct	13
2	M	76	Humphrey snapshot	Negative	L. tumor resection	252
3	F	68	Humphrey	Positive	R. occipital/temporal hemorrhage	18
4	F	45	Humphrey	Positive	L. posterior cerebral artery stroke	19
5	M	68	Humphrey snapshot	Negative	R. posterior cerebral artery stroke	24
6	M	54	Humphrey	Positive	L. posterior cerebral artery + cerebellar stroke	18
7	M	60	Humphrey	Positive	R. posterior cerebral artery stroke	6
8	M	35	Humphrey	Positive	L. occipital infarct	12
9	M	69	Humphrey	Positive	L. posterior cerebral artery stroke	19
10	M	28	Humphrey	Positive	L. occipital infarct	156
11	M	55	Humphrey	Negative	R. posterior cerebral artery stroke	36
12	F	37	Humphrey	Positive	L. posterior cerebral artery stroke	7
13	F	41	Humphrey	Positive	L. posterior cerebral artery stroke	12

### MRI Scanning Procedure

**FIGURE 1:**
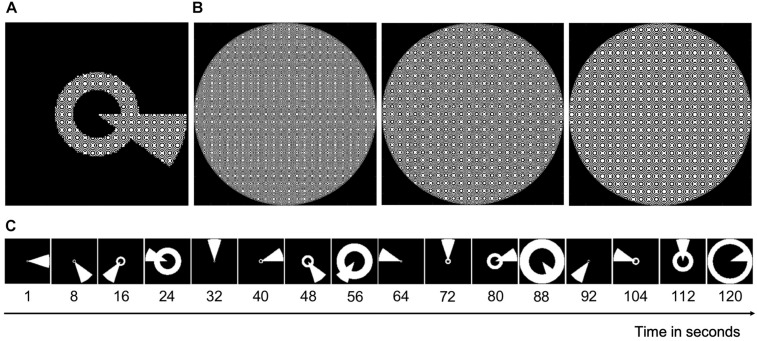
An illustration of the stimulus. **(A)** Image of the combined wedge-ring stimulus with the carrier (see **B**). **(B)** The stimulus carrier, a dynamic high-contrast pseudo-checkerboard pattern that varied in spatial frequency and phase. **(C)** Static binarized images of every 8th second of a full stimulus cycle of 120 s. During Scan 1, this cycle was repeated twice followed by a blank period of 48 s. During Scan 2, the cycle reversed, yet with the wedge starting again at 3 o’clock.

To reconstruct the VF for each participant, they took part in a VF mapping experiment during which we acquired two fMRI scans (each ∼5 min). The mapping stimulus shown to the participants was a simultaneous “wedge and ring” stimulus (Alvarez et al., 2015, see Figure 1A) with a dynamic high-contrast pseudo-checkerboard pattern (see Figure 1B). The stimulus radius had a VF coverage of 6.5° of visual angle. The wedge subtended 18° and the ring size varied with eccentricity following a logarithmic function. During Scan 1, the wedge started at 3 o’clock and rotated clockwise around a fixation point, while the ring expanded simultaneously starting from the most extracted position. During Scan 2, the wedge started at 3 o’clock and rotated counter clockwise, while the ring contracted simultaneously starting from the most eccentric position. Each 288 s scan consisted of two cycles of 120 s, in which the stimulus advanced every 2 s (i.e., 1 TR), followed by a blank period of 48 s. For patients P1 and P2, the stimulus was squeezed and thus did not cover the full 6.5° in the vertical dimension. For patient P1, the sequence of Scan 1 was repeated during Scan 2. See Figure 1C for an illustration of one stimulus cycle.

During the experiment, participants performed a simple attention task to control for central eye fixation. They were instructed to fixate at all times on the blue dot in the center of the screen and press a button on a response box when the dot changed color. An EyeLink 1000 eye tracker (SR Research Limited) was used to confirm fixation by recording eye movements.

### MRI Data Acquisition and Processing

Each participant underwent one or two MRI session(s) as part of a larger study at the Oxford Centre for Functional MRI of the Brain (FMRIB), Oxford, United Kingdom using a Siemens MAGNETOM Verio 3T MRI scanner^1^ with a 32-channel head coil. fMRI data were always collected in the same session, but the structural scan was sometimes from another session.

#### Anatomical Acquisition and Pre-processing

For each participant we acquired a high-resolution whole-head T1-weighted MPRAGE scan (voxel size = 1 mm isotropic, TE = 4.68 ms, TR = 2040 ms, field of view = 200 mm^2^, flip angle = 8°). The anatomical images were processed using the FreeSurfer segmentation tool (Dale et al., 1999) as implemented on brainlife.io, a free cloud platform for neuroscience data analysis, to obtain a gray-white matter segmentation. When necessary, this automatic segmentation was manually refined using the ITKGray segmentation software (Yushkevich et al., 2006).

In addition, the anatomical images were parcellated into 12 visual responsive cortical regions per hemisphere (i.e., V1, V2, V3, hV4, VO1, VO2, LO1, LO2, TO1, TO2, V3b, and V3a), using the neuropythy python library (Benson and Winawer, 2018) implemented on brainlife.io. For the main analysis, a single large visual cortex region of interest (“visual cortex ROI”) consisting of all twelve parcellated visual regions was constructed. In addition, a significantly smaller motion area hMT+ ROI was formed from the combination of TO1 and TO2 (Amano et al., 2009). By generating a separate VF reconstruction based on fMRI data within hMT+ only, we can investigate its specific contribution to a participant’s reconstructed VF. For secondary analyses of the MP data, the cortical regions were divided into an “early” visual cortex ROI consisting of V1 and V2 and an “extrastriate” cortex ROI which included the remaining regions.

#### Functional Data Acquisition and Pre-processing

For each participant, the two functional scans were acquired using echo planar imaging (EPI) (148 volumes; voxel size = 3 mm isotropic, 34 transverse slices, TR = 2000 ms, TE = 30 ms, flip angle = 90°). Four dummy volumes were acquired at the beginning of each scan in order for the magnetization to stabilize to a steady state. The total imaging time per scan was therefore approximately 5 min and a total of 10 min of data were used for the analysis.

Functional data were analyzed using VISTASOFT, a software package for analyzing (f)MRI data using MATLAB^2^. Pre-processing steps included slice-timing correction and a between and within scan motion correction (Nestares and Heeger, 2000). Time-series corresponding to the same stimulus cycles were averaged, as well as those corresponding to the blank period, to increase the signal:noise ratio of the data. The averaged time-series were aligned to the anatomical image and resampled to a 1 mm isotropic resolution using trilinear interpolation.

### Visual Field Mapping Techniques

We used two different fMRI-based VF mapping techniques: a conventional yet fast one (pRF mapping; Dumoulin and Wandell, 2008), and a more detailed yet computationally more demanding one (MP; Carvalho et al., 2020).

#### Conventional Population Receptive Field Mapping

**FIGURE 2:**
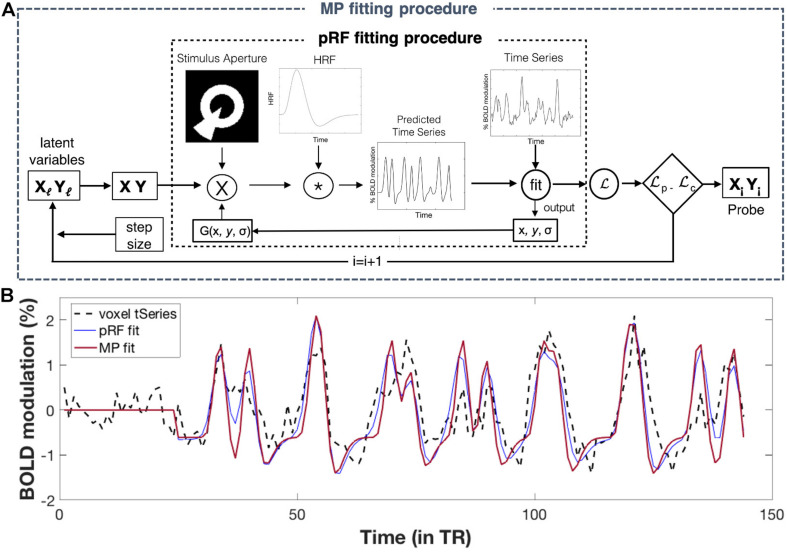
Population receptive field (pRF) and MP fitting: **(A)** Flowchart of fitting procedures. *Conventional pRF fitting procedure*: First, a two-dimensional Gaussian pRF is defined using three parameters: x and y (for its center location), and σ (for its width). The pRF response is predicted by calculating the overlap between the pRF (2D Gaussian) and the stimulus aperture. Next, this predicted response is convolved with an HRF function in order to obtain a time-series prediction for a particular voxel. For all possible pRFs, these predicted time-series are iteratively tested to fit a voxel its actual time-series. The optimal pRF properties correspond to ones that result in the best pRF fit (i.e., the one with the highest variance explained) per voxel. For a more detailed explanation of this fitting procedure, see Dumoulin and Wandell (2008). *MP fitting procedure*: The MP fitting procedure is based on the conventional pRF fitting procedure. Here, a Bayesian Markov Chain Monte Carlo (MCMC) sampling approach was used to fit a 2D Gaussian probe to voxels time-series. With this approach, the probe’s center location (x, y) is based on two latent variables and the width (σ) is small and fixed (here we used σ = 0.01°). Like the pRF procedure, a voxel’s predicted response was calculated and convolved with an HRF function before fitting it to the voxel’s actual response. The fit is done using likelihood, which allows the update of the latent variables (x, y), for the next iteration. The MCMC sampling approach therefore allowed for an efficient sampling of the VF, where the regions in visual space with better fits are more highly sampled. To this end, MP provides a single probe map per voxel, consisting of many probe locations with their corresponding variance explained, reflecting the sampling density of that particular voxel. For a more detailed explanation of this fitting procedure, see Carvalho et al. (2020) (Figure adapted from Carvalho et al. (2020); used with permission). **(B)** Example fits for a single voxel. Simulations of pRF- and MP fit to the actual time-series of a single voxel, using the pRF parameters of the conventional pRF and the MP best fit. The flat line at the start corresponds to the blank period. For more details on how to extract the pRF parameters for MP, see Supplementary Figure 2 of the Supplementary Material.

In the conventional pRF mapping technique, the response of a population of neurons (i.e., a voxel) is modeled to find its receptive field (RF). This is done by fitting a 2D-Gaussian to each voxel’s time-series with three free parameters: x0, y0, and σ. For the best model fit, these parameters correspond to the x and y coordinates of the preferred center location and the width of the voxel’s RF, respectively. For flowchart of the pRF fitting procedure, see Figure 2A. PRF modeling was performed for all voxels within our ROIs using the VISTASOFT toolbox in MATLAB and using SPM’s canonical hemodynamic response function (HRF). For examples of pRF derived eccentricity maps for both a patient and a control participant, see Supplementary Figure 1 of the Supplementary Material.

#### Micro-Probing

In MP, the response of a population (i.e., a voxel) or subpopulation of neurons (within a voxel) is modeled to find its RF. This is done by applying a large number of “micro-probes,” tiny 2D Gaussians with a small standard deviation (0.01°), to fit a voxel’s time-series while sampling across the entire stimulus space. The sampling of the visual space is done using Bayesian Markov Chain Monte Carlo (MCMC) approach, resulting in the regions of the visual space that best fit a voxel’s response being more densely sampled. For flowchart of the MP fitting procedure, see Figure 2A. A total of 10000 micro-probes were used to determine the VF sampling for every voxel within our ROIs. For computational benefits (parallel estimation), we divided both our visual cortex ROIs into three equally sized sub-ROIs. The MP approach resulted in one sampling density map for every voxel within our ROIs, with the probes weighted by their respective explained variance. Unlike the conventional pRF mapping, MP allows for the reconstruction of a detailed VF coverage of a single voxel, including the possibility of revealing multiple clusters of probes with high variance explained (VE) (i.e., pRFs) and of various shapes. Figure 2B shows an example of a pRF and MP fit for a single voxel.

### fMRI Based Visual Field Reconstruction

The two mapping techniques described above allow for a reconstruction of the VF by back-projecting the derived RF properties of all voxels within our ROI onto the VF. The resulting reconstruction–a coverage-map (CM)–reflects the VF sampling density of the modeled visual cortex ROIs. Since a high sampling density is likely to reflect reliable neural responses in the sampled VF location, this CM can indirectly reflect VF sensitivity of the ROIs.

#### pRF Based Visual Field Reconstruction

Only the pRF models with a minimum VE of 15% and with a maximum eccentricity of 6.5° were used for the VF reconstruction. For the hMT+ ROI this led to an average removal of 87.8% (SD = 8.1) and 90.6% (SD = 8.9) of the voxels for the controls and patients, respectively. Due to these high numbers of bad model fits, no reliable VF reconstructions could be made based on hMT+ data alone. Hence, only pRF-based VF reconstructions using the fMRI data of the entire visual cortex were made.

First, for each participant, a single CM was reconstructed for the left and right visual cortex ROI by summing the pRF models of the thresholded voxels (VE > 15%) within the ROI and by weighting them by their respective VE. Then, individual full-field CMs were created by averaging the CMs across hemispheres and by normalizing the resulting CM to a 0–1 range. Next, to correct for the high sampling density in the center of the VF, as a result of cortical magnification, and to make the VF reconstructions comparable across participants, the individual CMs were normalized using a normative VF map. For the patients, the normative map was the average of the normalized CMs of all controls. For the controls, the normative map was the average of the normalized CMs of all controls minus the one in question. For each participant, this resulted in a VF reconstruction for the visual cortex ROI denoting the normalized sampling density throughout the VF.

#### MP Based Visual Field Reconstruction

For each participant, the micro-probe maps for each voxel were first converted to a single CM (i.e., heat maps of a 26 × 26 bin grid weighted by the probes’ VE) and then summed across all the voxels in each ROI. In order to be included in the VF reconstruction, at least one bin of the CM had to have a minimum VE of 15%. For hMT+ this led to an average removal of 81.8% (SD = 1.1) and 93.3% (SD = 7.8) of the voxels for the controls and patients, respectively. Due to these high numbers of bad model fits, no reliable VF reconstructions could be made based on hMT+ data alone. Hence, as for the pRF technique, only MP-based VF reconstructions using the fMRI data from the visual cortex ROIs were made.

**FIGURE 3:**
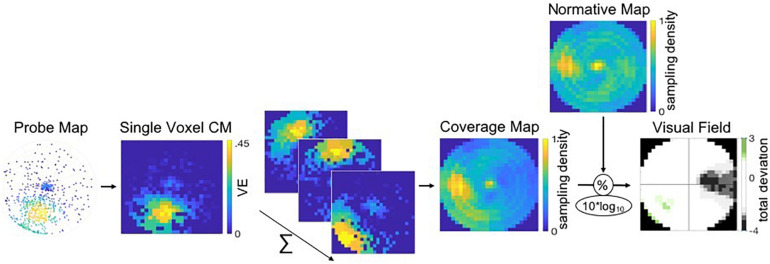
Pipeline of the VF, or coverage map, reconstruction of a patient using MP. Probe-maps are converted to single CMs per voxel. CM of voxels within a ROI are summed and CM across ROIs are averaged. Individual full-field CMs are normalized to a 0–1 scale and divided by a normative VF map. The resulting total deviation plot indicates whether the deviation is within normal limits (i.e., the 90% CI depicted in white) or below and above normal limits, as depicted by the gray and green tones, respectively.

Then, for each hemispheric sub-ROI, a CM was reconstructed by summing the CMs of the thresholded voxels within the ROI. Next, individual full-field CMs were created by averaging the CMs of all six sub-ROIs and by normalizing the averaged CM to a 0–1 range. Finally, this CM was normalized using a normative VF map, as described in section “MP Based Visual Field Reconstruction,” which resulted in a single CM for the visual cortex ROI denoting the total deviation of sampling density of the VF. For the illustration of the MP VF reconstruction pipeline, see Figure 3 (adapted from Carvalho et al. (2020); used with permission).

#### Comparison of Perimetric VF and fMRI Based VF

First, we converted the normalized CMs to a dB scale by taking the 10 × log_10_ of the sampling density values, making them comparable to the Total Deviation sensitivity plots (in dB) of the HFA perimetry test. Note that, from here onward, we use the term VF sensitivity to describe the VF coverage as derived with the fMRI techniques. Yet, strictly speaking, it reflects a relative sampling density which is not fully equivalent to the threshold sensitivity as determined using HFA.

We then plotted frequency distributions of the obtained VF sensitivities for the control, patients’ healthy and patients’ lesioned hemisphere, together with the 90% CI boundaries. To be able to visually and intuitively compare the reconstructed and perimetric VFs, we identified the 90, 96, 98, and 99% CI boundaries of the control VF sensitivity and scaled the color bar of the reconstructed VFs such that less than 5%, between 5 and 95% and more than 95% were visualized as below, within or above normal limits, respectively.

#### Comparison of pRF and MP Sensitivities

To test whether pRF and MP resulted in similar VF reconstructions, we compared the VF sensitivity estimates of both techniques. For this, we displayed the quantiles of the VF sensitivity distributions using a quantile-quantile plot, for the control, patients’ healthy and patients’ lesioned hemispheres. In case of similar sensitivities, the plot should appear approximately in line with a linear reference. Furthermore, to explore the relationship between the VF sensitivities, we made a scatter plot and fitted a polynomial curve using the least squares method.

## Results

**FIGURE 4:**
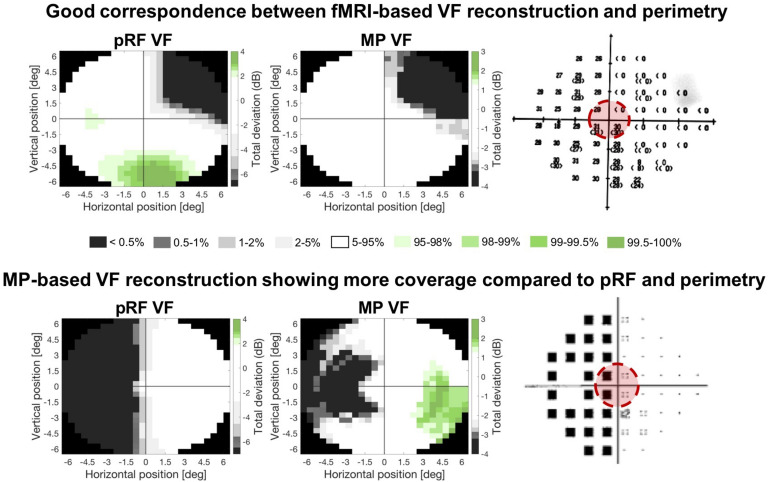
Comparison of the two fMRI-based VF reconstructions with the perimetric VF. Total deviation values are color coded according to the CI intervals of the Total Deviation distributions of the controls where white indicates the deviation to be within normal limits (i.e., the 90% CI) and the gray and green tones below (lower 5%) and above (upper 5%) normal limits, respectively. The zeros (upper) and black squares (lower) in the perimetric plots (panel on the right) indicate the VF locations that are considered blind. The red circle indicates the part of the VF that could be reconstructed with the fMRI-based approaches. Top row: example patient in whom both fMRI-based VF reconstructions show a high degree of correspondence to the perimetric VF plots. Bottom row: example patient with a high correspondence between the pRF-based VF reconstruction and the perimetric VF, while the MP-based VF reconstruction reveals a substantially larger VF coverage.

For all thirteen patients, visual inspection suggested a good correspondence of both fMRI-based VF reconstructions to the perimetric VF. For some patients, the fMRI-based VF reconstructions revealed sensitivity in parts of the VF that were considered as “blind” by perimetry. Furthermore, VF sensitivity is depicted in more detail and with higher sensitivities using MP, compared to pRF. Figure 4 shows the reconstructions and VF of two example patients (P11 and P13). For Patient 13 (top row), all three measures of the VF show excellent correspondence. In contrast, for Patient 11 (lower row) the MP derived VF is larger than the pRF derived VF.

### Distributions of VF Sensitivity

**FIGURE 5:**
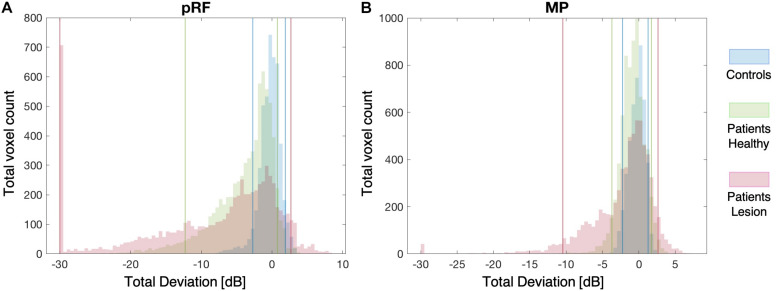
Distribution plots of VF Sensitivity. The distribution of the VF sensitivities of both the controls and patients for the pRF mapping **(A)** and the MP **(B)** techniques. The solid blue, green, and pink lines indicate the 90% boundaries of VF sensitivity for the controls, patients’ healthy and patients’ lesioned hemisphere, respectively.

Figures 5A,B show the distribution of VF sensitivities in the controls and the patients for the pRF and the MP technique, respectively. For the controls, 90% of the VF sensitivity fell within the range of −2.7 dB and +1.9 dB for the pRF mapping and −2.2 dB and +1.3 dB for the MP technique. In the healthy hemisphere of the patients, these boundaries corresponded to −12.3 dB and +0.7 dB for the pRF mapping and −3.7 dB and +1.7 dB for the MP technique. In the lesioned hemisphere, 90% of the VF sensitivity fell within a range of −30.0 dB and +2.7 dB for the pRF mapping and −10.5 dB and +2.6 dB for the MP technique.

Table 2 lists the VF sensitivities at the boundaries of the 90, 96, 98, and 99% CIs. One point to note is that in the lesioned hemisphere of the patients both the pRF and MP techniques identify a number of voxels that appear to have increased sensitivity relative to the control data, indicated by the positive red values in Figure 5. The reason for this apparent increase in neural sensitivity is unclear, but it could reflect enhanced ipsilateral activation of responsive voxels in the lesioned hemisphere to stimuli presented in the sighted field. This has previously been shown to be present in a study using a similar population (Ajina et al., 2015).

**TABLE 2:** CI boundaries of VF sensitivity.

Group	Technique		Confidence Interval [lower boundary; upper boundary]			
			90%	96%	98%	99%
*Controls*	pRF		[−2.7; +1.9]	[−4.6; +2.4]	[−5.6; +2.7]	[−6.5; +3.0]
	MP		[−2.2; +1.3]	[−2.7; +1.5]	[−2.9; +1.8]	[−3.2; +2.0]
*Patients*	pRF	Healthy Hemisphere	[−12.3; +0.7]	[−15.5; +1.5]	[−17.6; +1.9]	[−19.3; +2.7]
		Lesioned Hemisphere	[−30.0; +2.7	[−30.0; +4.1]	[−30.0; +5.5]	[−30.0; +6.2]
	MP	Healthy Hemisphere	[−3.7; +1.7]	[−5.0; +2.3]	[−6.1; +2.7]	[−8.8; +3.0]
		Lesioned Hemisphere	[−10.5; +2.6]	[−14.6; +3.6]	[−18.5; +4.4]	[−30.0; +5.2]

*The VF sensitivities at the boundaries of the 90, 96, 98, and 99% Confidence Intervals (CIs), as estimated by the pRF and MP techniques, for the control hemispheres and the healthy and lesioned hemispheres of the patient separately.*

### Good Correspondence Between fMRI Based VF Reconstructions and Perimetric VF

**FIGURE 6:**
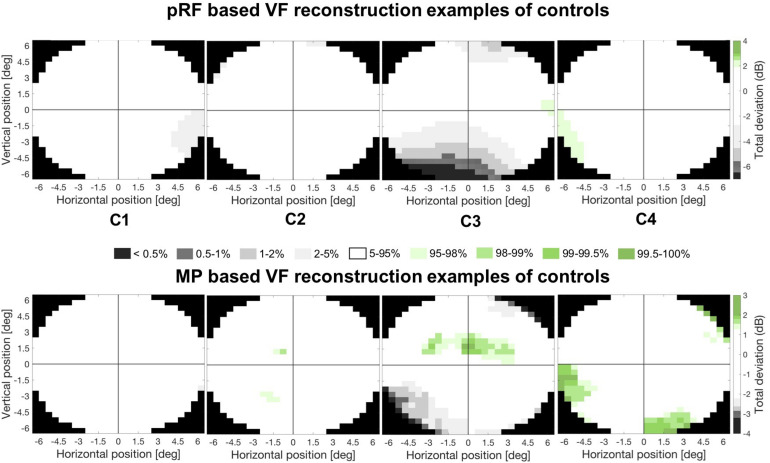
Example VF reconstructions. VF reconstructions for both the pRF mapping (upper panel) and MP technique (lower panel) for 4 control participants. In these plots, white pRF or MP-based coverage indicate VF sensitivity to be within normal limits (90% CI), the gray and green patches indicate a VF sensitivity below (lower 5%) or above (upper 5%) normal limits, respectively. Only the central 6.5° were reconstructed, reflecting the region of the VF stimulated during the VF mapping.

Figure 6 shows the pRF based (upper panel) and MP based (lower panel) VF reconstructions for four control participants. The vast majority of points in their VF reconstructions show activity within the normal range. However, C3 appears to show reduced activity in the lower field, but this is unlikely to reflect a “real” loss of visual sensitivity. Rather it could be explained by experimental issues such as reduced visibility at the edge of the stimulus. Across all controls, there were very few regions showing such reduced coverage.

**FIGURE 7:**
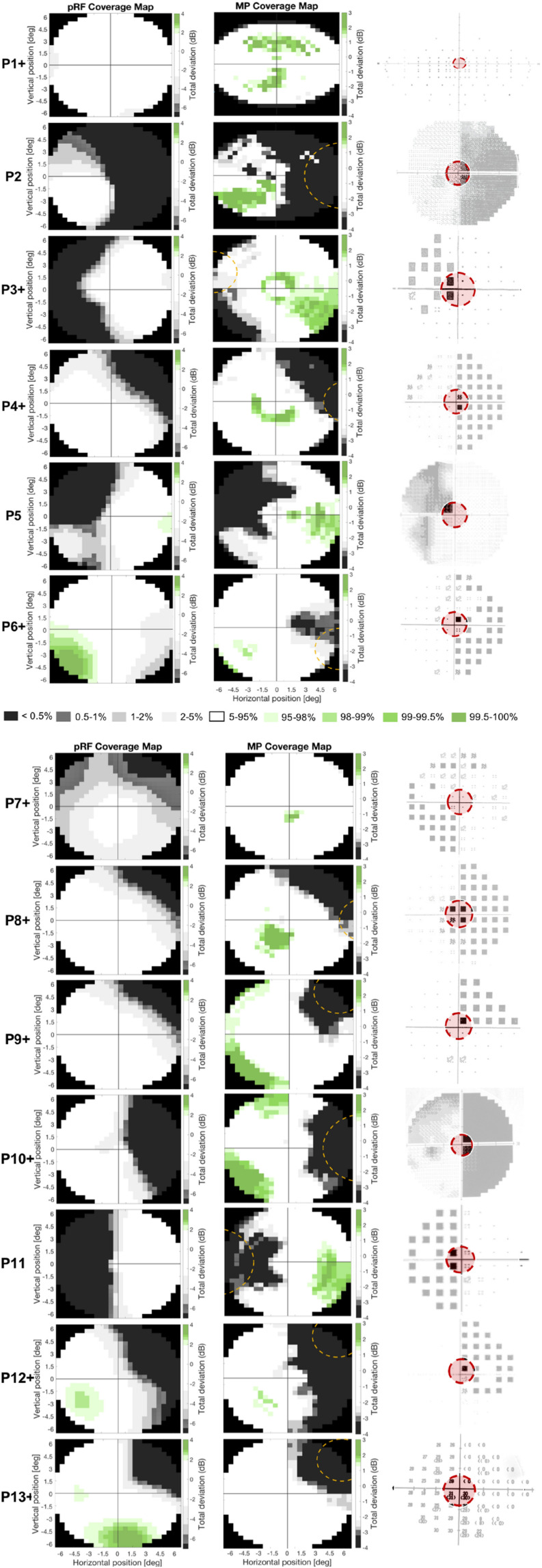
Comparison of the two different fMRI based VF reconstructions to perimetry. Figure shows the pRF- (left column) and MP-based VF reconstruction (middle column) and the perimetric VF (right column) for the thirteen patients. White pRF or MP-based coverage indicate VF sensitivity to be within normal limits (90% CI), the gray and green patches indicate a VF sensitivity below (lower 5%) or above (upper 5%) normal limits, respectively. The perimetric VF of P1 is measured with an Esterman VF test and denotes the VF locations where a presented stimulus was seen (circle) or missed (black square). The perimetric VFs of P2, P5, and P10 are measured with HFA and are graphical representations of the measured contrast sensitivity across the VF, where the darkness reflects lack of sensitivity, such that black is considered blind. The perimetric VFs of P13 were measured with HFA and have numerical representations of the measured contrast sensitivity across the VF, and lower values reflect lower sensitivity with zero is considered blind. The perimetric VFs of P3, P4, P6, P7, P8, P9, P11, and P12 are measured with HFA and are graphical representations of the Total Deviation of the measured contrast sensitivity across the VF, which means the more textured a pixel the more it deviates from normative data. Black pixels are considered blind. Dashed orange lines indicate stimulus location used in blindsight testing. The red circle in the perimetric VF indicates the VF coverage that could be measured with fMRI (i.e., the inner 6.5°), the part of the VF not covered with fMRI has faded out. The “+” after the patient number denotes the patients that were tested positively for blindsight.

Figure 7 shows the VF reconstructions of all patients based on pRF (left column) and MP (middle column). The right column shows the perimetric VF, with the dashed red circle indicating the VF stimulated by the VF mapping stimulus (i.e., 6.5°). In the VF reconstructions, white regions indicate a VF sensitivity within normal limits (i.e., the 90% boundaries of the control distribution) while the gray/black and green regions indicate below (<−2.73 dB for pRF or <−2.23 for MP) and above (>+1.87 dB for pRF or >+1.27 for MP) normal limits, respectively. The dashed orange line outlines the blindsight stimulus boundary in the ten patients whose test location overlapped with the VF mapping stimulus.

The perimetric VF image of P1 is an Esterman plot and denotes the VF locations at which the stimulus was either seen (circle) or missed (black square). The perimetric VF images of P3, P4, P6, P7, P8, P9, P11, and P12 are HFA’s Total Deviation probability plots. Total Deviation plots denote the probability that a given deviation in contrast sensitivity lies within the normal range for the patient’s age, with the textured pixels representing a probability of 5% or lower. The perimetric VF images of the remaining patients are also HFA plots but now denote a graphical representation (P2, P5, and P10), for which black corresponds to 0 dB (i.e., no sensitivity) and white to 41 dB (i.e., high sensitivity), or a numerical (P13) representation of the actual contrast sensitivity measured at each stimulus location.

For all patients, we found good correspondence between the fMRI-based VF reconstructions and the perimetric VF. In particular, this was the case for the reconstruction of the perimetric sighted parts of the VF (i.e., VF contrast sensitivity was within normal limits), that were also within normal limits based on the fMRI VF reconstructions. For the reconstruction of the perimetric blind parts of the VF (i.e., a contrast sensitivity below normal limits) we found, for some participants, that the fMRI techniques revealed a VF sensitivity within normal limits. In other words, for some patients, the fMRI-based VF reconstructions revealed normal sensitivity in parts of the VF which were classified as significantly impaired or even “blind” by perimetry.

### VF Sensitivity Estimated by MP Higher Than by pRF

**FIGURE 8:**
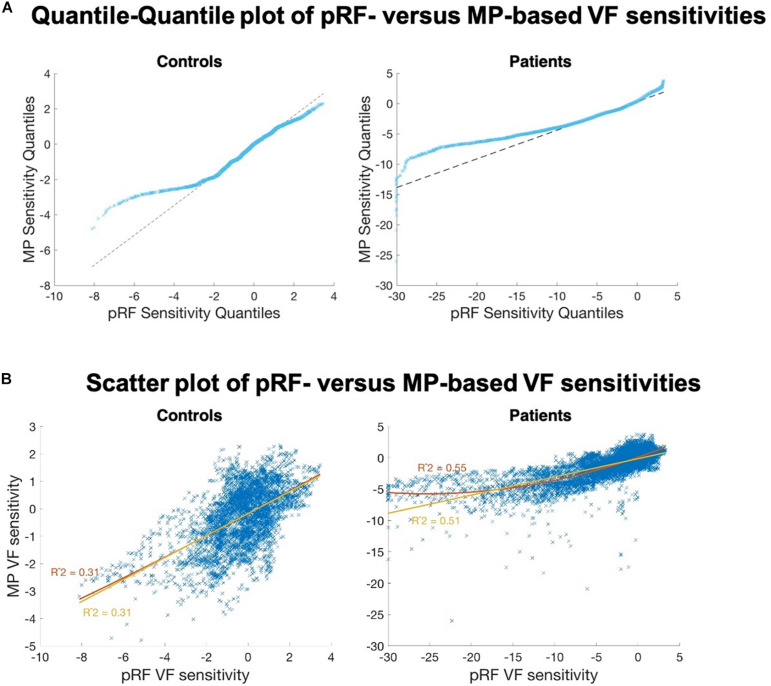
Comparison of the VF sensitivities as measured with the two fMRI approaches. **(A)** Quantile-Quantile plot of the VF sensitivities of pRF versus MP, for controls (left) and patients (right). Strong deviations from the linear reference line indicate that both distributions do not share the same distribution. For the negative estimates, in particular below <2 dB (controls) and <10 dB (patients), we found fewer negative estimates for MP than for pRF. **(B)** PRF VF sensitivities plotted against the MP VF sensitivities, for the controls (left) and the patients (right). The control data are linearly correlated. The patient data revealed a non-linear (*R*^2^ = 0.55, red curve) rather than a linear (*R*^2^ = 0.51, yellow line) relationship between the two VF sensitivity estimates.

The quantile-quantile plot showed that the VF sensitivities in patients estimated by the two techniques do not come from the same distribution (Figure 8–upper panel). Up to a VF sensitivity loss of 10 dB, the two techniques seem to share the same distribution, but then appear to diverge. The VF sensitivity quantiles of MP plateau while the corresponding values for the pRF continue to decrease. For the patients, this finding is illustrated by the non-linear relationship revealed by the scatter plot (Figure 8–lower panel); the two VF sensitivities are significantly more likely to be non-linearly (*R* = 0.55) than linearly related (*R* = 0.51; *z* = 3.34; *p* < 0.001). For the extreme negative VF sensitivities, i.e., a sensitivity loss of more than 15 dB, the estimated VF sensitivity losses are smaller for MP than for pRF.

In the VF reconstructions these differences are reflected in the presence of more details, i.e., small regions with higher VF sensitivities, in the MP compared to the pRF-based reconstructions. For patients P3, P4, P7, P11, and P12 the MP even revealed sensitivity in parts of the VF that remained undetected by the conventional pRF technique, as can be seen in Figure 7 and as was also illustrated in Figure 6.

### VF Coverage in Early Visual Cortex Compared to Extrastriate Regions

The data shown thus far was aggregated across all informative voxels in the visual cortex (visual cortex ROI). There are a number of neural substrates that may underlie residual function in patients with hemianopia, such as spared regions of V1 or extrastriate regions such as hMT+ or V4. The limited amount of data, as a result of the short scanning time, meant that there were not enough informative voxels to investigate individual visual areas. However, in an attempt to determine whether the neural activity corresponding to the blind region was due to spared early visual cortex (i.e., V1 and V2) or extrastriate regions (i.e., V3, hV4, VO1, VO2, LO1, LO2, TO1, TO2, V3b, and V3a), separate CMs were calculated for each of these large ROIs for the MP technique.

**FIGURE 9:**
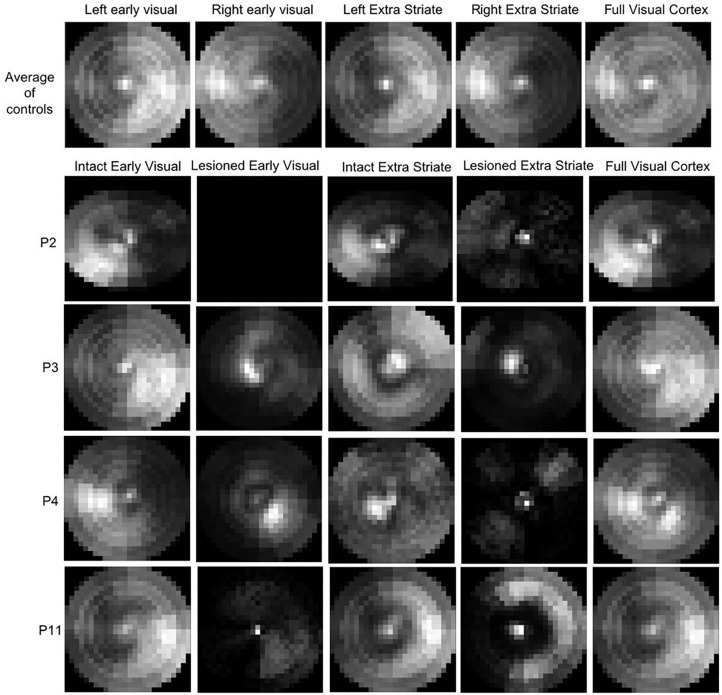
Coverage Maps for early (V1/V2) and extrastriate cortical regions averaged across the control (top row). The brighter the map, the higher the sampling density. P2, who is blindsight negative, shows very little contribution to the VF sampling by voxels in either early or extrastriate ROIs. P11, also blindsight negative, shows some contributing voxels in the sighted hemifield, but few in the blind field.

The top row of Figure 9 shows the average CMs across the controls. The ROIs for each hemisphere were analyzed separately to allow comparison of the hemisphere for which the CM is generated. Indeed, this is evident in the figure by the intensity of the CM in each hemifield. In terms of the relative contribution of the early and extrastriate areas, Table 3 shows that 46.4% ± 6.5% (mean ± s.d.) of informative voxels were from early visual cortex, and 53.6% ± 6.5% from extrastriate cortex, with no significant difference in their contribution.

**TABLE 3:**
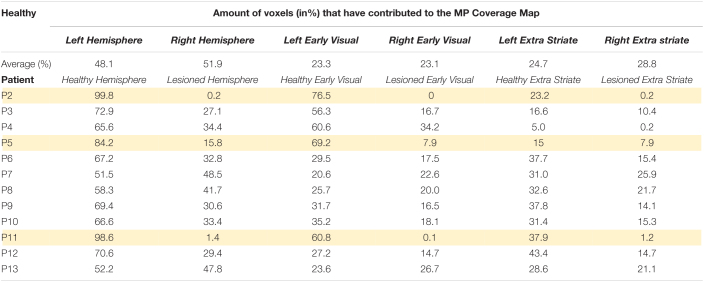
Location of voxels contributing to Coverage Maps based on MP.

*The three patients who were blindsight negative are highlighted. In each case the percentage is of the total contributing voxels across both ROIs and both hemispheres.*

The lower rows of Figure 9 show examples of CM for early and extrastriate regions in four patients, two blindsight positive (P3 and P4) and two blindsight negative (P2 and P11). The blindsight positive patients both show informative voxels from the lesioned hemisphere in the “blind” field, whereas in the blindsight negative patients the informative voxels from the lesioned hemisphere mainly represent the sighted field.

Table 3 shows the proportion of voxels contributing to CM in the lesioned and healthy hemispheres of the patients. Across all patients, there was a significantly higher proportion of informative voxels in the healthy hemisphere compared to the lesioned one (mean lesioned: 37.3%; mean healthy: 62.7%; *t* = 4.5; d.f. = 12; *p* < 0.001). This pattern was also evident when early and extrastriate visual areas were considered separately. In early visual cortex, the mean percentage of voxels meeting threshold criteria was 16.5% in the lesioned hemisphere which was significantly lower than the percentage of 41.7% in the healthy hemisphere (paired *t*-test: *t* = 3.5; d.f. = 12; *p* < 0.005). In extrastriate regions, the mean for the lesioned hemisphere (13.5%) was significantly lower than for the intact hemisphere (28.3%; *t* = 4.8; d.f. = 12; *p* < 0.0005). There was no difference in voxel contribution from early visual areas compared to extrastriate areas in either the lesioned or healthy hemisphere.

In terms of comparing blindsight positive and negative groups, there were only 3 patients who were blindsight negative, but the proportion of informative voxels in the lesioned hemisphere was considerably lower (5.8%) than in blindsight positive patients (37.3%; Mann-Whitney *U* = 0; *p* < 0.01).

### Effect of Stimulus Configuration

**FIGURE 10:**
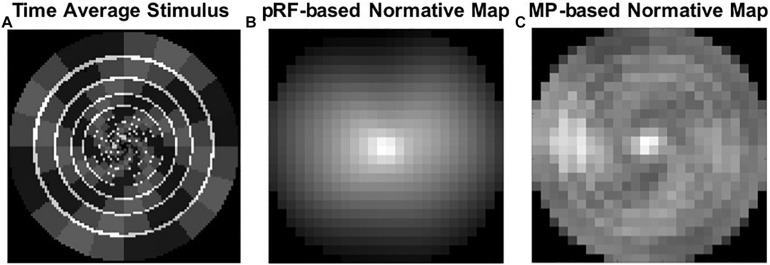
An effect of stimulus configuration. **(A)** A time average of the full stimulus presentation using a moving window of 12 TR. **(B)** PRF based normative map. **(C)** MP based normative map, which reflects the stimulus bias in VF stimulation.

Although we did not aim to investigate this, we observed a clear effect of stimulus configuration on the MP but not on the pRF estimates. This observation was most vivid in the normative VF map used for the normalization step during the VF reconstruction. Figure 10A shows a time average of the full stimulus presentation using a moving window of 12 TR (i.e., 24 s, comparable to the length of a typical HRF) and reveals that, over time, some parts of the VF are more stimulated (i.e., brighter segments) than others (i.e., darker segments). Such bias in VF stimulation is not reflected in the pRF (Figure 10B), but is strongly reflected in the MP based normative map (Figure 10C).

## Discussion

Using two different approaches to create fMRI-based VF reconstructions, we uncovered visually responsive sections in perimetrically blind regions of the VF in patients with hemianopia. This finding corroborates previous results based on conventional pRF mapping of V1 and hMT+ in hemianopia (Papanikolaou et al., 2014, 2019), which also showed the presence of neural activity in perimetrically blind regions of the VF. Compared to conventional pRF modeling, MP reveals more voxels with retained visual sensitivity, suggesting it is a more sensitive approach for VF reconstruction. Below, we discuss our results in more detail.

### Micro-Probing Reveals Larger Regions of Neural Activity Than pRF Mapping in Participants With Hemianopia

While the two approaches used in this study produced similar results in the sighted participants, the MP technique uncovered greater regions of responsive VF compared to the conventional pRF mapping in the patients.

Being a relatively new technique, MP provides a largely assumption-free approach to reconstructing the VF map from fMRI data (Carvalho et al., 2020, 2021). While it provides broadly equivalent results to standard pRF mapping, MP has the advantage that it can uncover and model multiple populations of RFs within a single voxel. This advantage is critical for studying participants with conditions such as albinism, where there is a bilateral representation of the VF in both hemispheres (Carvalho et al., 2020, 2021). In hemianopia, it may be that neural plasticity leads to more bilateral responses in hMT+ (Goebel et al., 2001) or that rehabilitation training changes the balance of activity in the two hemispheres (Henriksson et al., 2007; Larcombe et al., 2018). Thus, the MP approach that allows multiple locations to be mapped for a particular voxel may uncover sensitivity that would remain hidden when only a single modeled pRF can be assigned to a voxel.

One could argue that the larger regions of neural activity revealed by MP might be a result of computational error rather than a reflection of the true VF. We do, however, believe that the larger regions do reflect the true VF. MP has shown to be more conservative in estimating the pRF size than the conventional pRF technique, as well as more robust to the noisy signals (Carvalho et al., 2020). Therefore, if our results were biased by the MP technique, we would expect to obtain a smaller VF, not larger. Furthermore, MP has the advantage of providing a pRF coverage map (probe map) per voxel, which represents the pRF profile without any assumptions about its shape. The conventional pRF method assumes that the pRF shape has circular symmetry, which can lead to biases in the VF coverage maps at the border of the scotomas due to partially stimulated pRFs, i.e., the center of the pRF lies within the healthy VF and part of the pRF coverages the blind VF. This will result in biases at the border of the scotomas. The MP method is robust to these biases.

### The MP Estimates Are Less Negative Compared to the pRF Ones, for Areas With Considerable Loss of Sensitivity

When the sensitivity estimates are below 10 dB (i.e., the areas with great loss of sensitivity) the MP estimates are less negative than the pRF ones. One could argue that the MP approach is hitting a floor effect at this point, as if it is picking up more noise. We do, however, interpret these differences as a reflection of more VF coverage by the MP technique. First, MP has shown to be more robust to noisy signals (Carvalho et al., 2020) and second, if it were a general bias from the technique itself we expect this to be reflected as an increased sensitivity across the blind VF. Yet, the parts of the VF where the pRF revealed a strong deviation in sensitivity while the MP revealed a less strong deviation are along the border of the VF defect, regions likely to have residual visual sensitivity. We therefore suggest that these are areas with some level of visual function that cannot be detected by the pRF technique, but can be detected by MP.

### Stimulus Configuration Can Affect the Visual Field Map

One interesting finding, and an unexpected bycatch of the present study, was that MP allows reconstruction of the stimulus configuration from the BOLD activity. A time-average of this stimulus configuration with a time window of 24 s, equivalent to the length of the typical hemodynamic response of the human brain, revealed that this stimulus configuration results in unequal stimulation of the VF. MP was able to reveal this pattern, whereas the conventional pRF technique was not. Similarly, MP, but not pRF, was able to reveal the unstimulated parts of the VF in P1 and P2. These findings show that the MP technique is sensitive enough to detect such stimulation-biases. Moreover, it suggests that the conventional pRF estimates obtained with the combined wedge and ring stimulus are potentially biased by the stimulus configuration. Although a previous study on the effect of stimulus configuration on VF map properties showed that this simultaneous wedge and ring combination resulted in the highest pRF fit reliability (Alvarez et al., 2015), our present results suggest that the stimulus type should be carefully considered when aiming for fMRI-based VF reconstructions. In line with this, Infanti and Schwarzkopf (2020) showed that pRF mapping is sensitive to the protocol used and that the mapping sequence can bias its estimates. This higher sensitivity of MP, compared to the conventional pRF technique, favors the former over the latter for the reconstruction of the VFs.

### Effect of Contributing Voxels

The analysis considers the voxels that contribute to the coverage maps. However, it is important to interpret these numbers cautiously; three important issues should be considered. Firstly, larger lesions automatically result in fewer voxels that can contribute to the CMs. Secondly, the limited amount of pRF mapping data that was acquired (∼10 min) resulted in poor model fits which meant that we had to exclude many voxels, in particular for the higher order areas (for example as discussed for hMT+). Therefore, we cannot determine whether the removal of a large number of voxels with poor model fits were due to (1) a true lack of responsiveness or (2) a lack of reliable data. Finally, one should note that there is no lower limit of voxels to generate the coverage maps of the large ROIs, and therefore the CM in Figure 9 could have been generated based on a very small proportion of voxels. For example, the coverage for P11 as revealed by the MP but not by the pRF technique, includes a small proportion of voxels from the extrastriate areas of the lesioned hemisphere. Yet, this does not mean that the coverage is invalid or meaningless. Since the pRF technique assumes a single gaussian shaped RF (Dumoulin and Wandell, 2008) whereas MP fits many tiny probes (Carvalho et al., 2020), the latter technique has many more options of finding suitable VF locations that can explain each voxel’s activity based on the stimulus pattern. This may have particularly benefited the extrastriate areas.

### Correspondence Between Non-conscious Behavior and VF Reconstruction

Ten patients had an area of overlap of at least 4 deg^2^ between blindsight stimulus test locations and VF mapping in the central 6.5°, permitting comparison between MP and pRF VF reconstructions in locations tested behaviorally for preserved non-conscious vision. For both blindsight negative patients (P2 and P11), there was no appreciable difference in pRF or MP sensitivity in the blindsight test zone. Both patients showed reduced neural response outside normal limits (<1% CI).

Of the eight patients demonstrating blindsight, five patients (P4, P9, P10, P12, and P13) did not show sufficient fMRI signal to detect activity in the blindsight test region using either reconstruction technique. There are several reasons why this may be the case. It may be that our technique to normalize total deviation to controls was not sufficiently sensitive for small regions of retained activity that are comparably weaker than normal activity levels (Ajina and Bridge, 2018). Our scan time was also very short, and associated with poor model fits in extrastriate hMT+ voxels. This meant that it was not possible to reconstruct VF coverage based on hMT+ activity alone. If hMT+ is the primary region contributing to visual processing at motion blindsight locations, then it may not be surprising that activity was absent as hMT+ voxels did not contribute to the VF reconstruction.

Two blindsight positive patients (P6, P8) showed retained fMRI signal in the blindsight test zone using both MP and pRF techniques, albeit not completely normal. This is consistent with previous studies (Papanikolaou et al., 2014; Schneider et al., 2019; Sanchez-Lopez et al., 2020), and suggests that both methods can reveal preserved visual processing in hemianopia beyond SAP. Interestingly, the blindsight stimulus in both patients overlapped the border of preserved and absent signal, raising the possibility that fMRI sensitivity may simply reflect better resolution than perimetry at the border zones of field loss.

One patient with blindsight (P3) showed greater sensitivity in the blindsight test zone using MP compared to pRF reconstruction, including activity within the “normal” range. This is potentially very interesting, and suggests that where the reconstructed VF appears more sensitive than perimetry (e.g., P11 along the vertical meridian), it may be useful to test for preserved non-conscious vision. Together with the demonstration of greater sensitivity to stimulus configuration, this suggests that there are situations in which MP is more sensitive to both conscious and non-conscious VF reconstruction.

### Study Limitations

There are a number of limitations of the current study, both technical and relating to the specific population. The participants with hemianopia participated in an MRI study that included many different experiments to provide characterization of their neural responses. The pRF mapping experiment was one element of this study, and therefore only around 10 min of data were acquired. This contrasts with studies focused on pRF mapping which often acquire at least 30 min of high-resolution data (Benson et al., 2018). The relatively small amount of data resulted in lower signal:noise ratio, which meant that it was not possible to reliably map the size and location of individual pRFs. In turn, this means that individual visual areas and their specific contribution to the signal could not be identified. This issue is even more acute in patient populations when visual maps are abnormal. In future studies it will be important to identify the spared visual areas that may maintain neural sensitivity to the affected regions of the VF. Furthermore, knowing whether the maintained neural activity results from spared processing in the affected pathway or from processing in unaffected, yet functionally connected, pathways may be important. Since some extrastriate visual areas may have representations of both sides of the VF [e.g., the Medial Superior Temporal area (Huk et al., 2002) and Fusiform Face Area (Grill-Spector et al., 2017)], any feedback from these regions could lead to activity in areas of the visual cortex even if not activated by the retina via lesioned cortex. Therefore, investigating the origin of the neural activity and determining those extrastriate visual areas with preserved visual representation, each with their own specialized functions, may indicate the optimal type of training stimulus to boost residual vision. Interestingly, in the study of Papanikolaou et al. (2019) some patients showed neural activity in both area V1 and hMT+ in the absence of any residual visual ability. However, this does not preclude the potential for “reactivating” this region with rehabilitation. Indeed, using a much larger population of patients with hemianopia, Elshout et al. (2021) showed that regions of the VF that showed neural activity mapping using the pRF approach were more likely to show improvement following 80 h of visual training. However, since the neural activity was not quantified after the training, it is not clear whether training also enhanced the relevant neural activity. The study of Barbot et al. (2020) also found that the strength of V1 activity in perimetrically blind regions predicted the amount of improvement following training, although there was little change in activity after training. In a similar vein, Schneider et al. (2019) found that hemianopic patients showing fMRI activity in the visual cortex soon after their stroke showed less ganglion cell loss 6 months later. Understanding how this neural activity across the visual cortex relates to residual function may provide the basis for designing personalized rehabilitation programs that target spared visual pathways in patients.

In terms of the visual stimulus, the size of the VF that can be stimulated within the scanner was limited. The visual display for the current study subtended an eccentricity of 6.5°, which is only a small proportion of the entire VF. The VF deficits that are caused by hemianopia can affect any region, and therefore may not be within the small field of view visible within the scanner. Indeed, Figure 7 shows that in several of the cases presented here, the VF deficit was too peripheral to be detected by the fMRI scanning. For future studies, it would be worth investigating possible methods to increase the visible region within the scanner (Cornelissen et al., 1997; Greco et al., 2015; Elshout et al., 2021; Jolly et al., 2021).

Related to this, future studies could benefit from additional behavioral visual performance measures to confirm the presence of residual vision in the extended areas revealed by the fMRI techniques, in particular MP, as was performed by Elshout et al. (2021).

An additional issue is that the mapping stimulus used for the study did not stimulate the VF equally. As discussed earlier, this can be detected by the MP technique, but it is not optimal particularly since different patterns of stimulation can interact with VF deficits.

To fully understand how neural activity in different visual areas might relate to residual vision, or blindsight, it will be important to (i) acquire significant quantities of data to maximize signal:noise ratios, (ii) make the visible stimulus as large as possible to ensure it includes the VF deficit of all patients, (iii) ensure that the stimulus provides equal stimulation across the VF, and (iv) use psychophysical visual testing at the extended VF locations as revealed with the MP technique to quantify residual vision in these regions.

## Conclusion

In summary, fMRI can be used to identify responsive parts of the VF, that are not activated by the near-threshold lights used in perimetry, even with relatively little imaging data. This type of fMRI-based VF reconstruction may provide an important addition to standard perimetry techniques, particularly for identifying regions that may be amenable to rehabilitation programs to improve residual vision. The MP technique appears to provide additional sensitivity over conventional pRF mapping and may be particularly useful for investigating the abnormal visual system.

## Data Availability Statement

The raw data supporting the conclusions of this article will be made available by the authors, without undue reservation.

## Ethics Statement

The studies involving human participants were reviewed and approved by Oxfordshire Research Ethics Committee B. The patients/participants provided their written informed consent to participate in this study.

## Author Contributions

HH: conceptualization, methodology, validation, formal analysis, investigation, writing–original draft, and visualization. HB: conceptualization, methodology, data curation, resources, writing–review and editing. JC: methodology, validation, formal analysis, and writing–review and editing. FC: resources, writing–review and editing, and supervision. SA: conceptualization, methodology, data curation, resources, writing–review and editing, supervision, project administration, and funding acquisition. All authors contributed to the article and approved the submitted version.

## Conflict of Interest

The authors declare that the research was conducted in the absence of any commercial or financial relationships that could be construed as a potential conflict of interest.

## Publisher’s Note

All claims expressed in this article are solely those of the authors and do not necessarily represent those of their affiliated organizations, or those of the publisher, the editors and the reviewers. Any product that may be evaluated in this article, or claim that may be made by its manufacturer, is not guaranteed or endorsed by the publisher.

## Footnotes

1
www.healthcare.siemens.co.uk

2
https://github.com/vistalab/vistasoft

## Supplementary Material

Supplementary Figure 1 Population receptive field (pRF)-based eccentricity maps. Two visualizations of how output of the pRF mapping technique can be translated to eccentricity maps. The x and y Cartesian coordinates of the best pRF for each individual voxel were converted into polar coordinates, i.e., polar angle and eccentricity values. Here we projected the eccentricity values on an inflated hemisphere mesh. The upper two images display the eccentricity map for the left hemisphere of a control participant from a medial (left) and lateral (right) view. Similarly, the lower two images display the eccentricity maps for the left and lesioned hemisphere of a hemianopia patient. The case of the patient shows that also in the lesioned hemisphere we find cortical activity that partially represents the patient’s VF. The black outline corresponds to our visual cortex ROI, to which we limited our modeling computations. Projections have been thresholded for a variance explained of 15%.

Supplementary Figure 2 Extraction of MP derived pRF parameters. Left: initial probe map. Right: Thresholded probe map selecting the 30% probed with the highest VE (*k*-threshold). The remaining probes were clustered using a weighted cluster analysis. Finally, the pRF parameters of the resulting cluster, here four, were derived using a Gaussian mixture model. The derived parameters were x and y, for the pRF its center location, σ_1_ and σ_2_ and θ (Gaussian orientation, angle between σ_1_ and the *x* axis) allowing for an ellipsoidal Gaussian. Dashed blue circle outlines the pRF derived from the conventional pRF model, and the dashed red circle outlines the pRF derived from MP. For a more detailed description on this parameter extraction methods, see Carvalho et al. (2020).
