# Supplementary material for: Visual Field Reconstruction in Hemianopia Using fMRI Based Mapping Techniques
Source: Front Hum Neurosci. 2021 Aug 10;15:713114. doi: 10.3389/fnhum.2021.713114 (PMC8382851; doi:10.3389/fnhum.2021.713114)
Supplement: Supplementary Figure 1 — Population receptive field (pRF)-based eccentricity maps. Two visualizations of how output of the pRF mapping technique can be translated to eccentricity maps. The x and y Cartesian coordinates of the best pRF for each individual voxel were converted into polar coordinates, i.e., polar angle and eccentricity values. Here we projected the eccentricity values on an inflated hemisphere mesh. The upper two images display the eccentricity map for the left hemisphere of a control participant from a medial (left) and lateral (right) view. Similarly, the lower two images display the eccentricity maps for the left and lesioned hemisphere of a hemianopia patient. The case of the patient shows that also in the lesioned hemisphere we find cortical activity that partially represents the patient’s VF. The black outline corresponds to our visual cortex ROI, to which we limited our modeling computations. Projections have been thresholded for a variance explained of 15%. [file Image_1.pdf]

Supplementary Material

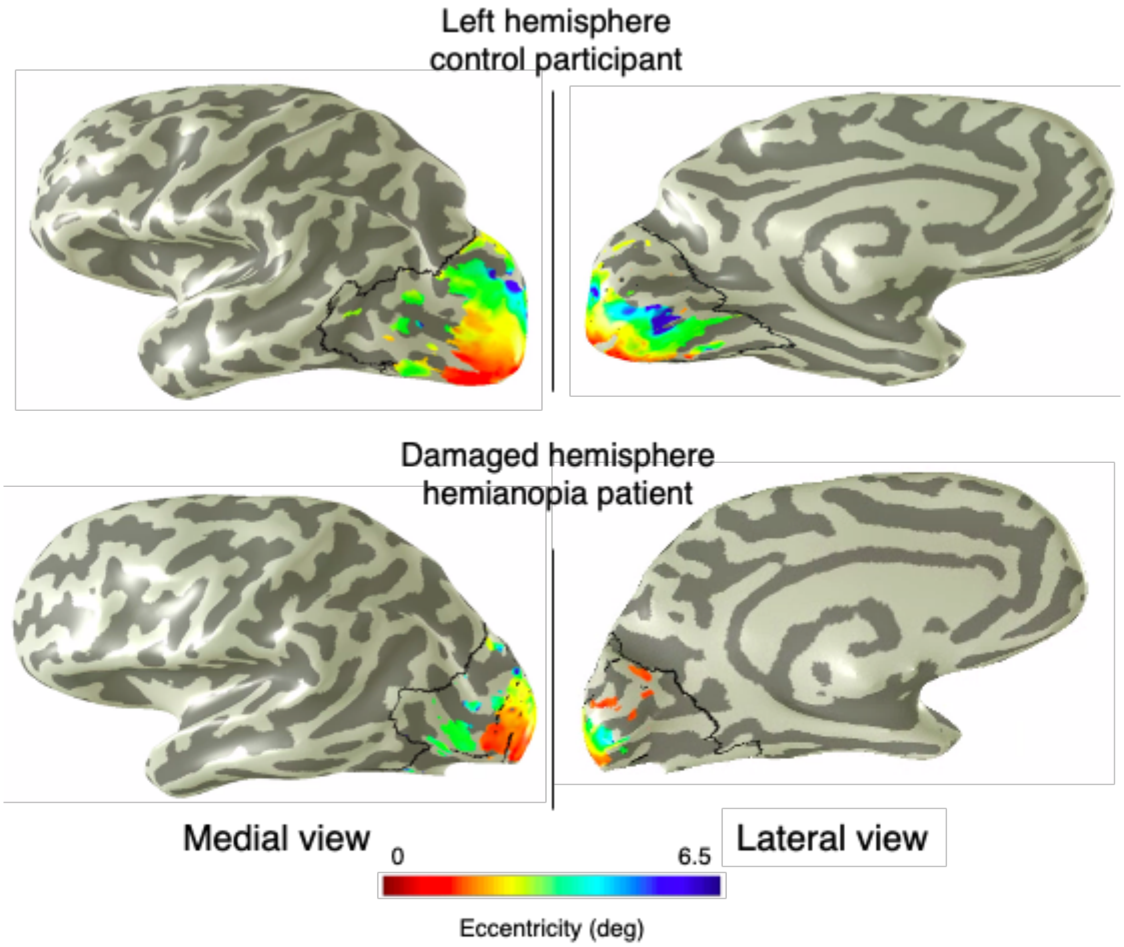

Figure I pRF-based eccentricity maps. Two visualisations of how output of the pRF mapping technique can be translated to eccentricity maps. The x and y Cartesian coordinates of the best pRF for each individual voxel were converted into polar coordinates, i.e., polar angle and eccentricity values. Here we projected the eccentricity values on an inflated hemisphere mesh. The upper two images display the eccentricity map for the left hemisphere of a control participant from a medial (left) and lateral (right) view. Similarly, the lower two images display the eccentricity maps for the left and lesioned hemisphere of a hemianopia patient. The case of the patient shows that also in the lesioned hemisphere we find cortical activity that partially represents the patient's VF. The black outline corresponds to our visual cortex ROI, to which we limited our modelling computations. Projections have been thresholded for a variance explained of 15%.
