# Supplementary material for: Visual Field Reconstruction in Hemianopia Using fMRI Based Mapping Techniques
Source: Front Hum Neurosci. 2021 Aug 10;15:713114. doi: 10.3389/fnhum.2021.713114 (PMC8382851; doi:10.3389/fnhum.2021.713114)
Supplement: Supplementary Figure 2 — Extraction of MP derived pRF parameters. Left: initial probe map. Right: Thresholded probe map selecting the 30% probed with the highest VE (k-threshold). The remaining probes were clustered using a weighted cluster analysis. Finally, the pRF parameters of the resulting cluster, here four, were derived using a Gaussian mixture model. The derived parameters were x and y, for the pRF its center location, σ1 and σ2 and θ (Gaussian orientation, angle between σ1 and the x axis) allowing for an ellipsoidal Gaussian. Dashed blue circle outlines the pRF derived from the conventional pRF model, and the dashed red circle outlines the pRF derived from MP. For a more detailed description on this parameter extraction methods, see Carvalho et al. (2020). [file Image_2.pdf]

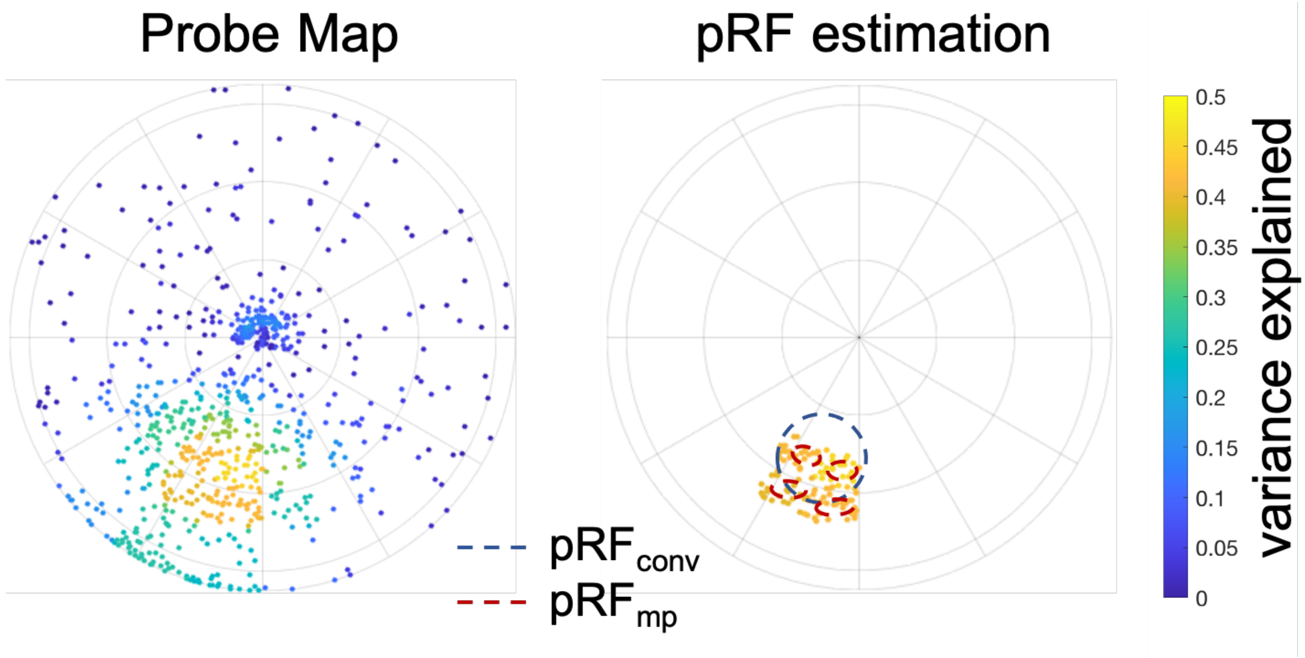

Figure II Extraction of MP derived pRF parameters. Left: initial probe map. Right: Thresholded probe map selecting the 30% probed with the highest VE (*k*-threshold). The remaining probes were clustered using a weighted cluster analysis. Finally, the pRF parameters of the resulting cluster, here four, were derived using a Gaussian mixture model. The derived parameters were x and y, for the pRF its center location, and σ_1 and σ_2 allowing for an ellipsoidal Gaussian. Dashed blue circle outlines the pRF derived from the conventional pRF model, and the dashed red circle outlines the pRF derived from MP. For a more detailed description on this parameter extraction methods, see Carvalho et al. 2020.
